# Prediction of future risk of any and higher-grade prostate cancer based on the PLCO and SELECT trials

**DOI:** 10.1186/s12894-022-00986-w

**Published:** 2022-03-26

**Authors:** Jonathan A. Gelfond, Brian Hernandez, Martin Goros, Joseph G. Ibrahim, Ming-Hui Chen, Wei Sun, Robin J. Leach, Michael W. Kattan, Ian M. Thompson, Donna Pauler Ankerst, Michael Liss

**Affiliations:** 1 Department of Population Health Sciences, Mail Code 7933, 7703 Floyd Curl Drive, San Antonio, TX 78229-3900 USA; 2 Department of Biostatistics, University of North Carolina, Chapel Hill, NC USA; 3 Department of Statistics, University of Connecticut, New Haven, NC USA; 4 Biostatistics Program, The Fred Hutchinson Cancer Research Center, Seattle, WA USA; 5 Department of Urology and Mays Cancer Center, University of Texas Health at San Antonio, San Antonio, TX USA; 6 Department of Quantitative Health Sciences, Cleveland Clinic, Cleveland, OH USA; 7 CHRISTUS Santa Rosa Hospital – Medical Center, San Antonio, TX USA; 8 Departments of Mathematics, Life Sciences, Technical University of Munich, Munich, Germany

**Keywords:** Prostate cancer, High grade prostate cancer, Risk prediction, Five-year risk

## Abstract

**Background:**

A model was built that characterized effects of individual factors on five-year prostate cancer (PCa) risk in the Prostate, Lung, Colon, and Ovarian Cancer Screening Trial (PLCO) and the Selenium and Vitamin E Cancer Prevention Trial (SELECT). This model was validated in a third San Antonio Biomarkers of Risk (SABOR) screening cohort.

**Methods:**

A prediction model for 1- to 5-year risk of developing PCa and Gleason > 7 PCa (HG PCa) was built on PLCO and SELECT using the Cox proportional hazards model adjusting for patient baseline characteristics. Random forests and neural networks were compared to Cox proportional hazard survival models, using the trial datasets for model building and the SABOR cohort for model evaluation. The most accurate prediction model is included in an online calculator.

**Results:**

The respective rates of PCa were 8.9%, 7.2%, and 11.1% in PLCO (n = 31,495), SELECT (n = 35,507), and SABOR (n = 1790) over median follow-up of 11.7, 8.1 and 9.0 years. The Cox model showed higher prostate-specific antigen (PSA), BMI and age, and African American race to be associated with PCa and HGPCa. Five-year risk predictions from the combined SELECT and PLCO model effectively discriminated risk in the SABOR cohort with C-index 0.76 (95% CI [0.72, 0.79]) for PCa, and 0.74 (95% CI [0.65,0.83]) for HGPCa.

**Conclusions:**

A 1- to 5-year PCa risk prediction model developed from PLCO and SELECT was validated with SABOR and implemented online. This model can individualize and inform shared screening decisions.

**Supplementary Information:**

The online version contains supplementary material available at 10.1186/s12894-022-00986-w.

## Background

Prostate cancer (PCa) is the most prevalent non-skin cancer in men in the United States [1]. Rates of PCa diagnosis may be decreasing but are most affected by screening practices, modulated by recommendations from groups such as the US Preventative Services Task Force (USPSTF) [2, 3]^,^. In 2012, the USPSTF recommended against use of prostate-specific antigen (PSA) for annual screening but later modified this decision to include shared-decision making [4, 5].

Clinical decision-making related to prostate cancer screening is challenging due to a lack of consistent guidance and the need for personalized decisions based on risk, a concept poorly understood by patients and many physicians [6, 7]. Noting that prostate-specific antigen (PSA) screening is a preference-sensitive decision, patients make screening decisions based upon their personal perceived risk along with the preferences and biases of their healthcare provider. Patients may understand risk better if they had numeracy skills regarding baseline and relative risk to understand what factors were related to their risk [8, 9]. Multiple clinical variables including family history, race, and abnormal digital rectal examination are predictive of PCa and may influence the decision to undergo prostate biopsy [10]. The value of these variables in selecting the screening interval is not well established [11].

Effective screening should involve patient input and be more frequent when the risk of developing PCa is higher and less frequent in lower-risk individuals [12]. We created a future PCa risk calculator that would help patients and providers develop a personalized screening schedule. We evaluated risk variables in two large controlled prostate cancer trials that included PSA screening: PLCO (Prostate Lung Colon Ovarian Screening Trial) and SELECT (Selenium and Vitamin E Cancer Prevention Trial) [13, 14]. We then evaluated predictions in the San Antonio Biomarkers of Risk (SABOR) [15] cohort as an independent validation.

## Methods

### Populations

The PLCO was a National Cancer Institute (NCI) sponsored trial designed to determine the impact of annual PSA screening for six years on cancer-related mortality and secondary endpoints in men ages 55–74. Men were recommended for prostate biopsy if their digital rectal exam (DRE) was suspicious, or their serum PSA exceeded 4 ng/mL. The trial was completed in 2006 [16, 17].

SELECT was an NCI sponsored, multicenter prostate cancer prevention trial. Enrollment began in 2001 and ended in 2004. Over 35,000 men, age 50 and older with PSA less than 4 ng/mL were randomized to receive placebo, selenium, vitamin E, or both selenium and vitamin E. Annual PSA and DRE testing were performed. Median follow-up was 5.5 years. The study arm receiving vitamin E had a significantly increased risk of PCa [18].

The SABOR prostate cancer study is an NCI sponsored multi-ethnic cohort of more than 3,880 healthy men from San Antonio and South Texas, recruited to evaluate biomarkers of PCa risk [15]. Study enrollment began in 2000; between 2000 and 2010, subjects had annual PSA visits and since 2011, men with PSA < 1.0 ng/ml were assessed less frequently.

### Selection criteria

We evaluated the PLCO, SELECT and SABOR cohorts at study entry for the following variables: age, race, digital rectal exam (DRE, suspicious for cancer or not), body mass index (BMI, kg/m^2^), benign prostatic hypertrophy (BPH) (present/absent), PSA, and first-degree family history. SELECT eligibility criteria excluded men with PSA of > 4 ng/ml and abnormal DRE at entry. We therefore restricted the analysis set in PLCO and SABOR to PSA < 4 ng/ml and to ages between 55 and 75 years to avoid bias due to men at higher than typical risk who may have had undiagnosed PCa at baseline. The subset of SABOR cohort was reduced from 3880 men 1790 men by restricting to men with a minimum of 30-day follow-up time and to those having additional information on both free PSA and food frequency questionnaire surveys at baseline.

### Statistical analysis

Baseline covariates were compared among cohorts using chi-squared tests for categorical variables and ANOVA for continuous variables. We treated age as a continuous predictor and log-transformed PSA. A grade was missing or masked in approximately 50% of PCa diagnoses for the PCLO and SELECT cohorts, and therefore multiple imputation with ten iterations was used for high grade PCa (HGPCa, Gleason > 7) analyses. To smooth the incidence curve, we imputed the time of occurrence ten times within the multiple imputation procedure as a randomly occurring between 0 and 6 months prior to the actual diagnosis time. All predictive models averaged over the imputations and the imputations were taken into account for computation of all standard errors. All data analyses were performed using R v3.5 + (Vienna, Austria). All statistical tests were two-sided at the 0.05 level.

### Predictive modeling

We created a prediction model using both SELECT and PLCO with the Cox proportional hazards model, deep neural network (DNN) survival models [19, 20], random survival forest (RF) survival models [21], and Fine-Gray competing risk models [19, 20] to distinguish high and low grades. The variables in the Cox models were selected based upon the Akaike’s Information Criterion, and an analysis of the effects of these variables is given in a supplement. We internally validated all prediction models for any PCa and HGPCa by splitting data from the PLCO and SELECT equally into training and test sets while balancing for study and cancer status. Similarly, the PLCO and SELECT survival models were used to estimate 5-year risk for the SABOR cohort. The predictions from the PLCO and SELECT models were combined using a weighted average where the weights are equal to the logistic regression prediction of study membership using the prediction variables as covariates. We constructed a web-application for suggested screening intervals based on a patients individual risk trajectory and a subjective risk tolerance. The 1-year risk represents annual screening and serves as an anchor for the patient to gauge future risk. For example, if a patient had a risk tolerance of 2% then and his 1, 2, and 3-year risk of cancer are 1.0%, 1.7% and 2.2%, then he should be screened at 2-years to avoid crossing his 2% risk-tolerance. The user sees that his risk increases incrementally each year. Patients with a higher risk tolerance or lower risk would have extended screening intervals. In this way, we suggest tailored screening times based upon risk factors and risk tolerance.

### Validation criteria

We assessed the accuracy of prediction using both discrimination and calibration. For discrimination, we calculated the C-index. Calibration was assessed by plotting the Kaplan–Meier observed risk against quantiles of the predicted risk [22]. The combined PLCO and SELECT data were used to calibrate the best model defined by the C-index using logistic regression with linear and quadratic terms. This model was then applied, with no additional calibration to the SABOR cohort for external validation.

## Results

The PLCO trial (n = 31,495) and SELECT (n = 35,507) contributed over 65,000 men combined to the build and test models, while SABOR contributed data from 1790 men for validation (Table 1).

**Table 1:** Baseline characteristics of the three studies used in the analysis

Variable	PLCO		SELECT	SABOR	SMD
n	31,495		35,507	1790	
Age (mean (SD))	62.47 (5.27)		62.93 (6.80)	58.68 (9.63)	0.35
African American = yes (%)	1279 (4.1)		5256 (14.8)	173 (9.7)	0.25
BMI (mean (SD))	27.61 (4.19)		28.59 (4.60)	28.17 (4.80)	0.15
Family history of cancer = yes (%)	2299 (7.4)		5929 (16.7)	424 (23.7)	0.31
Cancer diagnosis = yes (%)	2798 (8.9)		2550 (7.2)	198 (11.1)	0.09
Cancer grade (%)					0.28
Low-grade cancer	935 (33)		1756 (69)	169 (85)	
High-grade cancer (Gleason > 7)	375 (13)		146 (6)	22 (11)	
Missing grade	1488 (53)		648 (25)	7 (4)	
DRE = suspicious (%)	2082 (6.6)		3 (0.0)	295 (16.5)	0.44
BPH = yes (%)	6463 (20.6)		5334 (15.0)	626 (35.0)	0.31
Baseline PSA (mean (SD))	1.30 (0.88)		1.35 (0.94)	1.16 (0.79)	0.15
Follow-up years (median (IQR])	11.7 [9.6, 12.9]		8.1 [6.1, 10.3]	9.02 [4.76, 13.16]	0.54
PCa follow-up (median (IQR])	6.7 [3.5, 9.5]		4.2[2.3, 6.0]	5.62 [3.17, 8.32]	0.49
No PCa follow-up (median (IQR])	12.5 [10.5, 12.9]		8.4[6.5, 10.4]	9.74 [5.28, 13.44]	0.60

All omnibus *p*-values were less than 0.001. SMD is the average standardized mean difference for pairwise comparisons

Median follow-up time was 11.7 and 8.1 years in PLCO and SELECT, respectively (*p* < 0.001). Relative to SELECT, the PLCO cohort was slightly younger (62.5 vs. 62.9 years, *p* < 0.001), and had lower PSA at baseline (1.30 ng/ml vs. 1.34 ng/ml, *p* < 0.001). The PLCO also had less frequent family history of PCa than SELECT (7.4% vs. 16.7%, *p* < 0.001), but SABOR had the highest rate at 23.7%. The PLCO cohort had fewer African American men than SELECT (4.1% vs. 14.8%) and a higher rate of PCa diagnosis (8.9% vs. 7.2%, *p* < 0.001) than SELECT while SABOR had the highest rate at 11%. SABOR also had a substantially higher rate of HGPCa at 1.2% than SELECT (0.4%) or PLCO (1.2%).

**Fig. 1:**
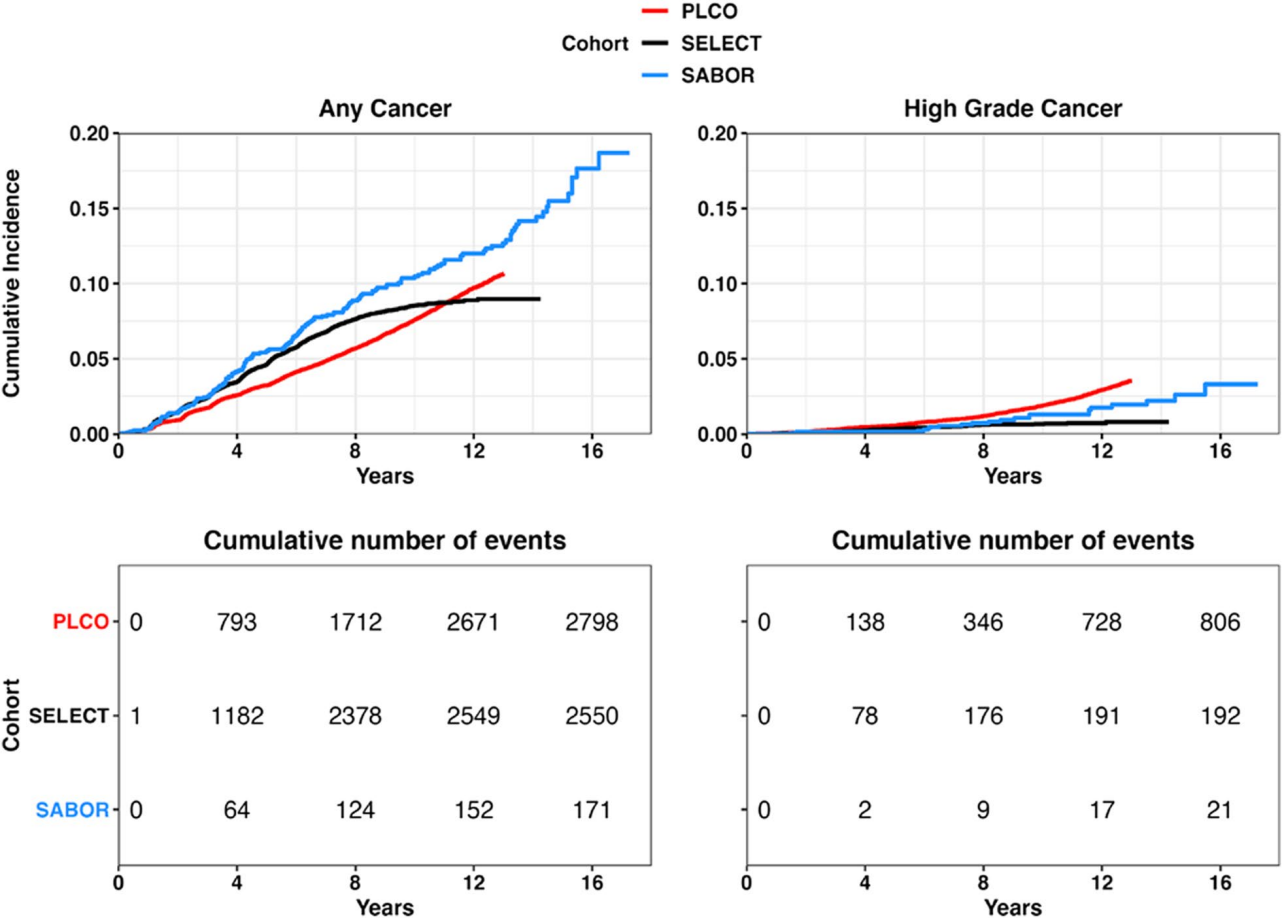
Time to event data for PLCO, SELECT, and SABOR studies

For PLCO and SELECT, Fig. 1 displays Kaplan–Meier PCa incidence over time: the proportional hazards model for time-to-any PCa for both PLCO and SELECT trials is summarized in Table 2.

**Table 2:** Cox proportional hazard models by study prior to AIC evaluation

Study	Variable	Hazard ratio	CI 95%	*P* value
PLCO	Age	1.01	(1, 1.02)	0.08
	Age and PSA interaction	0.89	(0.85, 0.94)	< 0.001*
	BMI	1.01	(1, 1.02)	0.07
	BPH	0.88	(0.81, 0.97)	0.01*
	DRE	1.64	(1.46, 1.85)	< 0.001*
	Family history of cancer	1.43	(1.27, 1.61)	< 0.001*
	Finasteride use	0.69	(0.6, 0.79)	< 0.001*
	Log (PSA)	5.52	(5.15, 5.93)	< 0.001*
	African American	1.37	(1.15, 1.63)	< 0.001*
	African American and BMI interaction	1.05	(0.89, 1.23)	0.58
SELECT	Age	1.01	(1, 1.02)	0.03*
	Age and PSA interaction	0.83	(0.79, 0.88)	< 0.001*
	BMI	1	(0.99, 1.01)	1
	BPH	0.84	(0.75, 0.93)	0.001*
	Family history of cancer	1.72	(1.58, 1.88)	< 0.001*
	Finasteride use	0.98	(0.77, 1.25)	0.88
	Log (PSA)	5.3	(4.92, 5.71)	< 0.001*
	African American	1.29	(1.15, 1.44)	< 0.001*
	African American and BMI interaction	1.15	(1.04, 1.27)	0.01*

The bold and * denotes *P*-value < 0.05

The key variables predicting the increasing risk of PCA were age, PSA, DRE, family history, and African American race. In SELECT, BMI was associated with time-to-PCA for African Americans (HR 1.15, *p* = 0.01) but not for men of other races. In the PLCO trial, BMI was less associated with time-to-PCa (HR 1.01, *p* = 0.07), and this association was not specific to African American men (HR 1.05, *p* = 0.58). The results of the competing risk-models for low- and high-grade PCa are similar and given in Additional file 1: Tables S2 and S3.

**Fig. 2:**
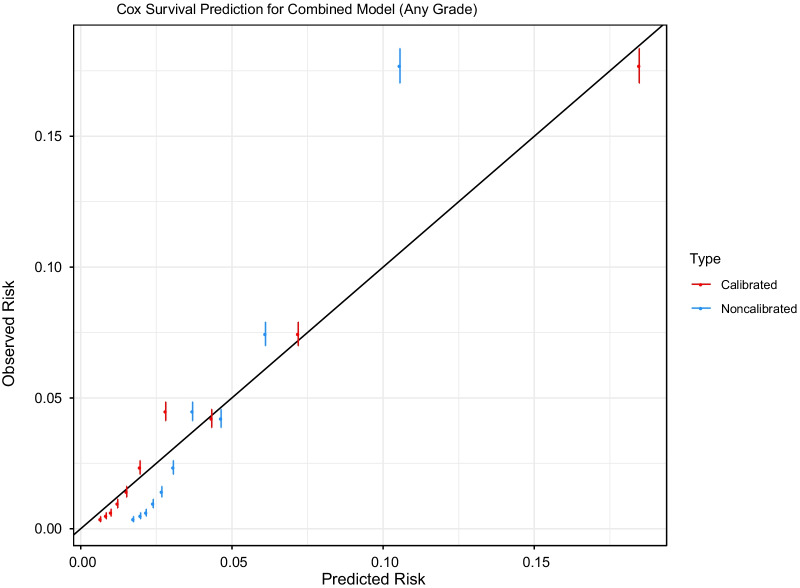
Calibration plot for risk of any prostate cancer from a combined model fit to the PLCO and SELECT training samples and applied to the PLCO and SELECT test samples, showing observed versus predicted risk for each risk decile. Predictions are shown pre/post calibration

**Fig. 3:**
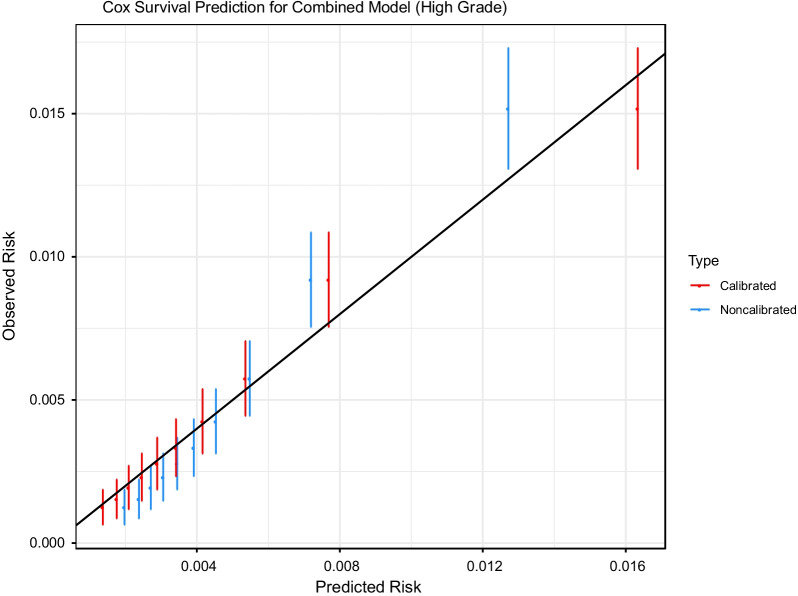
Calibration plot for risk of high-grade prostate cancer from a combined model fit to the PLCO and SELECT training samples and applied to the PLCO and SELECT test samples, showing observed versus predicted risk for each risk decile. Predictions are shown pre/post calibration

The Cox model outperformed the RF and DNN models in almost all cases so was used for the development of the risk prediction model (Additional file 1: Table S4). Adjustment for the competing risk of low-grade diagnosis in the analysis of HGPCa did not change outcomes and hence was not used. For any PCa, the SELECT and PLCO Cox models had an external (SELECT predicting PLCO and vice versa) prediction accuracy of C-index 0.78 (95% CI [0.77, 0.79]) and 0.79 (95% CI [0.78, 0.80]), respectively. For HGPCa, the SELECT and PLCO Cox models had an external prediction accuracy of C-index 0.72 (95% CI [0.7, 0.74] and 0.74 (95% CI [0.7, 0.79]), respectively. Using the combined model fit to 50% of the PLCO and SELECT trials and evaluated on the remaining 50% participants narrowly had the best prediction overall, but more so in the prediction of HGPCa, which may have resulted from a pooling of information about smaller event numbers in the separate trials. The Cox model closely approximates the observed risk within a few percentage points (Figs. 2 and 3). We produced a web-accessible calculator at (https://gelfondjal.shinyapps.io/apps/) that provides 1- to 5-year predictions with combined SELECT and PLCO model predictions. We provided an illustration of this in the Supplementary Figure. The application computes the suggested follow-up intervals for patients in the test samples of PLCO and SELECT in Supplementary Table 5. This table shows the time for which a certain risk tolerance is not exceeded for developing PCa of any grade. For example, with a 2% risk tolerance a majority (75%) of patients would not needed to before 2-years.

**Fig. 4:**
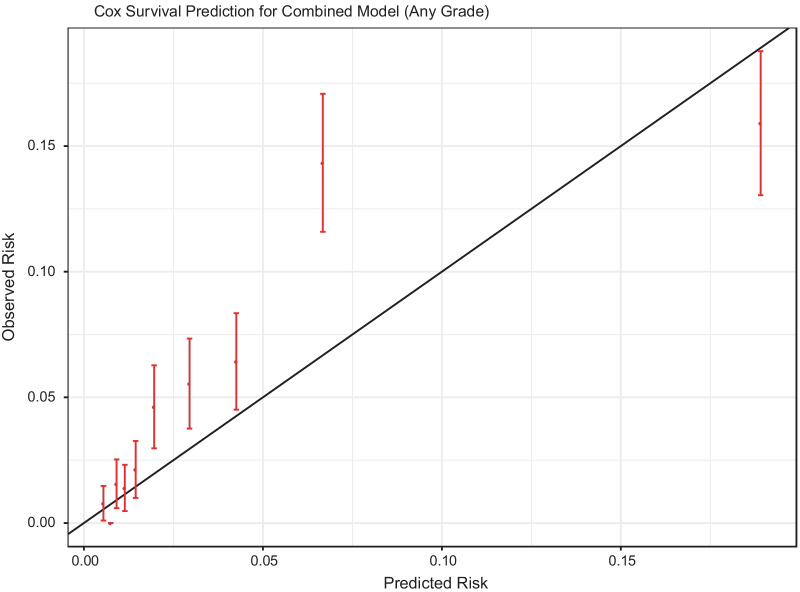
Calibration plot for risk of any prostate cancer from a combined model fit to the PLCO and SELECT training samples and applied to the SABOR dataset, showing observed versus predicted risk for each risk decile. Predictions were calibrated only to the training set

We evaluated predictions within the independent SABOR study, for any PCa, the SELECT, PLCO, and combined Cox models had a validation prediction accuracy of 0.73 (95% CI [0.7, 0.76]), 0.77 (95% CI [0.73, 0.8]), and 0.76 (95% CI [0.72, 0.79]), respectively in Supplementary Table 6. For HGPCa, the SELECT, PLCO, and combined Cox models had a validation prediction accuracy of C-index 0.74 (95% CI [0.65, 0.84]), 0.73 (95% CI [0.63, 0.83], and 0.74 (95% CI [0.65, 0.83]), respectively. The calibration curve for any PCa is shown in Fig. 4. The PCa risk calibration curve differed significantly from equivalence of observed and predicted risk (chi-squared test, *p* < 0.001), but the predictions for any PCa risk were reasonably close to the observed risk. However, the SABOR cohort only had 22 high-grade PCa cases, which prevented calibration from being evaluated. The SABOR overall observed risk of high-grade PCa at 5 years was low at 0.34% compared to calibrated overall predicted risk of 0.48%.

## Discussion

Many recommendations related to screening and monitoring for preventable/treatable diseases are espoused based on general population rates of disease. The ideal method for both prevention and early diagnosis would incorporate the risk characteristics of the *individual patient*, the natural history of the disease (i.e., the speed of disease development would dictate how early the diagnosis must be made to affect a cure), and the patient preferences. By developing an individualized assessment of disease risk, a personalized schedule of testing could be established. Similarly, with the established ability of the five-alpha reductase inhibitor finasteride to reduce a man’s risk of prostate cancer, this risk assessment can allow a patient to make an informed decision about the risk–benefit of PCa chemoprevention.

Strategically increasing the screening intervals would lower the frequency of screening and reduce screening-related risks such as psychological stress, financial toxicity, and overtreatment. Informing patients of risks due to screening results may cause anxiety and depression. Prostate biopsies prompted by screening results cause pain and carry the risk of infection. Positive biopsies precipitate overtreatment of low-risk cancers causing morbidity (pain, impotence, chemotherapy, and radiation effects) and mortality as well as unnecessary healthcare costs. Given these screening harms, methods have been proposed to extend the screening interval [23] based on cost-effectiveness and risk stratification [24]. Heijnsdikj et al. [25] proposed a model based on microsimulation of prostate cancer mortality that risk stratified upon PSA. These methods did not consider precise patient factors such as race or family history, nor do these methods explicitly incorporate patient risk tolerance. For example, men who have relatively long or short life expectancy might have different risk tolerances that affect screening decisions.

This study enabled the development of an online tool that incorporates these risk variables. For some patients whose risk is high, a conversation between the physician and patient may lead to annual of PSA testing. On the other hand, for most patients, the generally low risk of diagnosis could allow for a less-frequent testing schedule. We have previously found that patients with a PSA < 1.0 ng/mL could even consider a 10-year frequency of PSA testing due to the low risk of prostate cancer development [26].

## Limitations

There are limitations to this study. Both the timing of prostate cancer risk and the nature of clinical risk affect screening decisions. This prediction model for PCa incidence may be less important to patients than the future risks of PCa mortality and symptom burden. Also, particular treatment regimens in SELECT or screening regimens in PLCO could impact risk estimation. Given that vitamin E increased PCa, this would likely lead to a slight overestimation of risk. Estimates of 5-year risk are dependent on patient compliance with annual screening; for example, if a patient delays screening by six months to 5.5 years, then cancer would not be detected at 5-years, which could result in an underestimation of risk. The imputation assumption of missing-at-random is salient as grade was missing 53% of the cases for PLCO; this could lead to biased estimation for high-grade disease. In SELECT, missing grade was associated with African American race (18% vs. 14%, *p* = 0.02), longer mean follow-up time (5.8 vs. 3.8 years, *p* < 0.001), and lower PSA (2.1 vs. 2.4 ng/mL, *p* < 0.001). In PLCO, missing grade was associated with higher age (64 vs. 62 years, *p* < 0.001), BPH (24% vs. 20%, *p* = 0.01), and longer mean follow-up time (7.1 vs. 6.0 years, *p* < 0.001). However, we demonstrated that the model was accurate in predicting grade when grade was masked, and further exploration of competing risks of low- versus high-grade is warranted.

Another limitation is that differential propensity for biopsy and the precision of biopsy technology may bias model predictions. Factors that decrease the rate of biopsy may reduce the rate of PCa diagnosis and result in underestimated risk. New biomarkers may alter the rate of detection, and MRI guided biopsies could decrease the detection of clinically insignificant PCa [27] which would lower the harms of screening. Other risk factors such as genotype, were not considered and play a significant role in some men although the accuracy of prediction given addition genetic information was similar to results reported here [28, 29]. Finally, the less complex Cox model outperformed RF and DNN models, but this was likely due to nearly linear effects, model overfitting, and small event numbers for high-grade PCa. Larger sample sizes or alternative machine learning approaches could improve prediction.

## Conclusions

In three studies, encompassing 68,792 patients, we confirmed that a group of widely available clinical variables can predict a man’s 5-year risk of prostate cancer including tumor grade. Predictive variables for high-grade and low-grade PCa are different. The clinical prediction tool developed for this study can be helpful in determining the frequency of PSA testing and can guide conversations related to prostate cancer prevention. This methodology can be further studied in shared decisions with patient populations and with other risk factors, biomarkers, and genetic measures.

## Supplementary Information

**Additional file 1.** Supplementary tables and figures.

## Data Availability

The data that support the findings of this study are available from the SWOG Network (SELECT) and the National Cancer Institute Cancer Access System (PLCO) but restrictions apply to the availability of these data, which were used under license for the current study, and so are not publicly available.

## Abbreviations

PSA: Prostate specific antigen
PLCO trial: Prostate Lung Colon Ovarian screening trial
SELECT: Selenium and Vitamin E Cancer Prevention Trial
SABOR study: San Antonio Center of Biomarkers of Risk for Prostate Cancer
