# Supplementary material for: Prediction of future risk of any and higher-grade prostate cancer based on the PLCO and SELECT trials
Source: BMC Urol. 2022 Mar 26;22:45. doi: 10.1186/s12894-022-00986-w (PMC8966358; doi:10.1186/s12894-022-00986-w)

**Supplementary Table 1. Logistic Regression of high grade (Gleason>7) status.**

| **Study** | **Variable** | **Odds Ratio** | **95% CI** | **P-Value** |
| --- | --- | --- | --- | --- |
| **PLCO** | Time to Diagnosis | 1 | (0.95, 1.06) | 0.86 |
|  | Age | 1.04 | (1.02, 1.06) | **< 0.001*** |
|  | BMI | 1.02 | (1, 1.04) | 0.09 |
|  | log(PSA prior to High Grade Cancer) | 2.88 | (2.6, 3.19) | **< 0.001*** |
|  | Lag High Grade Cancer | 0.81 | (0.75, 0.87) | **< 0.001*** |
|  | African American | 1.41 | (1.06, 1.88) | **0.02*** |
|  | BPH | 0.96 | (0.81, 1.14) | 0.64 |
|  | DRE | 1.39 | (1.09, 1.77) | **0.01*** |
|  | Age and PSA Interaction | 0.97 | (0.9, 1.05) | 0.47 |
|  | African American and BMI Interaction | 1 | (0.77, 1.3) | 0.99 |
|  | Family History of Cancer | 1.25 | (0.98, 1.59) | 0.07 |
|  |  |  |  |  |
| **SELECT** | Time to Diagnosis | 0.84 | (0.8, 0.89) | **< 0.001*** |
|  | Age | 1.07 | (1.04, 1.09) | **< 0.001*** |
|  | BMI | 1.02 | (0.99, 1.06) | 0.17 |
|  | log(PSA prior to High Grade Cancer) | 0.75 | (0.72, 0.78) | **< 0.001*** |
|  | Lag High Grade Cancer | 0.41 | (0.34, 0.48) | **< 0.001*** |
|  | African American | 0.94 | (0.61, 1.44) | 0.77 |
|  | BPH | 0.87 | (0.58, 1.29) | 0.49 |
|  | Age and PSA Interaction | 1.03 | (0.98, 1.1) | 0.24 |
|  | African American and BMI Interaction | 0.91 | (0.64, 1.29) | 0.6 |
|  | Family History of Cancer | 1.58 | (1.12, 2.22) | **0.01*** |

* P-value < 0.05

**Supplementary Table 2. Competing Risk model predictors for High Grade cancer.**

| **Study** | **Variable** | **Hazard Ratio** | **95% CI** | **P-value** |
| --- | --- | --- | --- | --- |
| **PLCO** | Age | 1.06 | (1.04, 1.08) | **< 0.001*** |
|  | BMI | 1.02 | (1, 1.04) | **0.05*** |
|  | log(PSA) | 2.59 | (2.25, 2.97) | **< 0.001*** |
|  | African American | 1.95 | (1.46, 2.62) | **< 0.001*** |
|  | BPH | 0.9 | (0.76, 1.08) | 0.3 |
|  | DRE | 1.42 | (1.1, 1.83) | **0.01*** |
|  | Age and PSA Interaction | 0.9 | (0.84, 0.98) | **0.02*** |
|  | African American and BMI Interaction | 1.04 | (0.82, 1.32) | 0.73 |
|  | Family History of Cancer | 1.14 | (0.87, 1.5) | 0.36 |
|  | Finasteride use | 0.98 | (0.76, 1.26) | 0.88 |
|  |  |  |  |  |
| **SELECT** | Age | 1.05 | (1.01, 1.09) | **0.002*** |
|  | BMI | 1.03 | (0.99, 1.07) | 0.09 |
|  | log(PSA) | 3.29 | (2.4, 4.5) | **< 0.001*** |
|  | African American | 1.38 | (0.86, 2.2) | 0.18 |
|  | BPH | 0.65 | (0.41, 1.04) | 0.07 |
|  | Age and PSA Interaction | 0.91 | (0.75, 1.11) | 0.4 |
|  | African American and BMI Interaction | 0.92 | (0.65, 1.31) | 0.63 |
|  | Family History of Cancer | 1.45 | (1, 2.1) | **0.05*** |
|  | Finasteride use | 1.68 | (0.8, 3.54) | 0.17 |

* P-value < 0.05

**Supplementary Table 3. Competing Risk model predictors for Low Grade cancer.**

| **Study** | **Variable** | **Hazard Ratio** | **95% CI** | **P-value** |
| --- | --- | --- | --- | --- |
| **PLCO** | Age | 0.97 | (0.95, 0.99) | **< 0.001*** |
|  | BMI | 1 | (0.98, 1.02) | 0.63 |
|  | log(PSA) | 7.85 | (6.84, 9) | **< 0.001*** |
|  | African American | 1.06 | (0.82, 1.37) | 0.65 |
|  | BPH | 0.88 | (0.78, 0.99) | **0.04*** |
|  | DRE | 1.65 | (1.38, 1.97) | **< 0.001*** |
|  | Age and PSA Interaction | 0.99 | (0.92, 1.07) | 0.82 |
|  | African American and BMI Interaction | 1 | (0.79, 1.27) | 1 |
|  | Family History of Cancer | 1.54 | (1.31, 1.8) | **< 0.001*** |
|  | Finasteride use | 0.58 | (0.47, 0.72) | **< 0.001*** |
|  |  |  |  |  |
| **SELECT** | Age | 1 | (0.98, 1.02) | 0.45 |
|  | BMI | 1 | (0.98, 1.02) | 0.6 |
|  | log(PSA) | 5.53 | (5.01, 6.1) | **< 0.001*** |
|  | African American | 1.27 | (1.13, 1.43) | **< 0.001*** |
|  | BPH | 0.85 | (0.76, 0.96) | **0.01*** |
|  | Age and PSA Interaction | 0.84 | (0.77, 0.9) | **< 0.001*** |
|  | African American and BMI Interaction | 1.19 | (1.05, 1.33) | **0.003*** |
|  | Family History of Cancer | 1.75 | (1.59, 1.93) | **< 0.001*** |
|  | Finasteride use | 0.9 | (0.69, 1.19) | 0.45 |

* P-value < 0.05

**Supplementary Table 4. Prediction Accuracies of the Random Forest and Cox Models. 95% Confidence intervals denoted by [,].**

| **Grade** | **Validation Set** | **Training Set** | **C-index RF** | **C-index Cox** | **P-value** |
| --- | --- | --- | --- | --- | --- |
| Any | SELECT | PLCO | 0.76 [0.75,0.78] | 0.79 [0.78,0.8] | **< 0.001*** |
|  |  | SELECT | 0.76 [0.75,0.78] | 0.79 [0.78,0.8] | **< 0.001*** |
|  |  | Combined | 0.77 [0.76,0.78] | 0.79 [0.78,0.8] | **< 0.001*** |
|  | PLCO | PLCO | 0.75 [0.74,0.77] | 0.77 [0.76,0.78] | **< 0.001*** |
|  |  | SELECT | 0.73 [0.72,0.75] | 0.78 [0.77,0.79] | **< 0.001*** |
|  |  | Combined | 0.76 [0.75,0.77] | 0.77 [0.76,0.78] | **0.04*** |
| Gleason>7 | SELECT | PLCO | 0.71 [0.65,0.76] | 0.74 [0.7,0.79] | **0.01*** |
|  |  | SELECT | 0.7 [0.65,0.74] | 0.74 [0.69,0.79] | **0.02*** |
|  |  | Combined | 0.71 [0.66,0.76] | 0.75 [0.7,0.8] | **0.01*** |
|  | PLCO | PLCO | 0.67 [0.64,0.69] | 0.72 [0.7,0.75] | **< 0.001*** |
|  |  | SELECT | 0.64 [0.62,0.67] | 0.72 [0.7,0.74] | **< 0.001*** |
|  |  | Combined | 0.67 [0.64,0.69] | 0.72 [0.7,0.75] | **< 0.001*** |

* P-value < 0.05

**Supplementary Table 5. Suggested screening intervals for risk tolerance 1-5% for any cancer for patients in the test sets of PLCO and SELECT cohorts. The interval represents the time interval prior to the cancer risk exceeding the risk tolerance. The cells represent the numbers of participants and the (%) for each year.**

|  | **Years** | **1%** | **2%** | **Risk Tolerance**  **3%** | **4%** | **5%** |
| --- | --- | --- | --- | --- | --- | --- |
| Interval |  | N=33501 | 33501 | 33501 | 33501 | 33501 |
| Years (%) | < 1 | 8556 (25.5) | 2023 ( 6.0) | 429 ( 1.3) | 99 ( 0.3) | 54 ( 0.2) |
|  | 1 | 13830 (41.3) | 6211 (18.5) | 3254 ( 9.7) | 1847 ( 5.5) | 903 ( 2.7) |
|  | 2 | 10982 (32.8) | 7039 (21.0) | 5091 (15.2) | 3087 ( 9.2) | 2234 ( 6.7) |
|  | 3 | 125 ( 0.4) | 8907 (26.6) | 4411 (13.2) | 3932 (11.7) | 2678 ( 8.0) |
|  | 4 | 8 ( 0.0) | 7907 (23.6) | 5669 (16.9) | 3363 (10.0) | 3271 ( 9.8) |
|  | > 5 | 0 ( 0.0) | 1414 ( 4.2) | 14647 (43.7) | 21173 (63.2) | 24361 (72.7) |

**Supplementary Table 6. Prediction Accuracies of Cox Models. 95% Confidence intervals denoted by [,].**

| **Grade** | **Validation Set** | **Training Set** | **C-Index** |
| --- | --- | --- | --- |
| **Any** | SABOR | PLCO | 0.77 [0.73,0.8] |
|  |  | SELECT | 0.73 [0.7,0.76] |
|  |  | Combined | 0.76 [0.72,0.79] |
| Gleason>7 | SABOR | PLCO | 0.73 [0.63,0.83] |
|  |  | SELECT | 0.74 [0.65,0.84] |
|  |  | Combined | 0.74 [0.65,0.83] |

**Supplementary Figure.** Screenshot of future risk web application. The patient chooses a risk tolerance (Here 2%, dashed red line). The patients risk for any prostate cancer is shown on the vertical axis at screen times from 1 to 5 years on the horizontal axis.


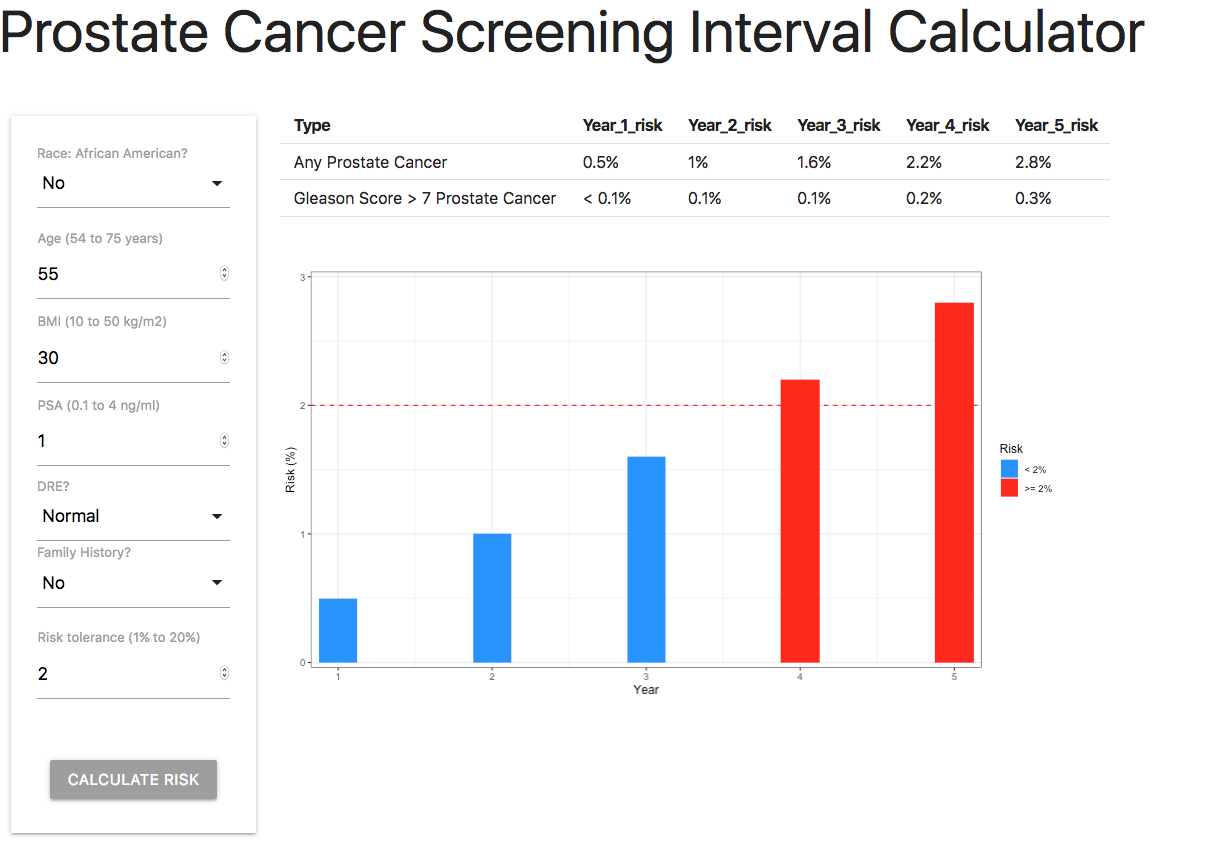

Supplement: Supplementary file 1 — Additional file 1. Supplementary tables and figures. [file 12894_2022_986_MOESM1_ESM.docx]
